# Epithelial-mesenchymal transition mediates anoikis resistance and enhances invasion in pleural effusion-derived human lung cancer cells

**DOI:** 10.3892/ol.2013.1108

**Published:** 2013-01-07

**Authors:** PREEDAKORN CHUNHACHA, VIROTE SRIURANPONG, PITHI CHANVORACHOTE

**Affiliations:** 1 Department of Pharmacology and Physiology, Faculty of Pharmaceutical Sciences; Chulalongkorn University and The King Chulalongkorn Memorial Hospital, Bangkok 10330, Thailand; 2 Cell-based Drug and Health Products Development Research Unit; Chulalongkorn University and The King Chulalongkorn Memorial Hospital, Bangkok 10330, Thailand; 3 Department of Medicine, Division of Medical Oncology, Faculty of Medicine, Chulalongkorn University and The King Chulalongkorn Memorial Hospital, Bangkok 10330, Thailand

**Keywords:** Thai, lung cancer, epithelial-mesenchymal transition, anoikis

## Abstract

Epithelial-mesenchymal transition (EMT) is implicated in cancer pathological processes, particularly cancer invasion and metastasis. The present study demonstrated that EMT was critical for the metastasic potential of lung cancer cells isolated from a patient. P1 primary lung cancer cells were found to exhibit increased anoikis resistance compared with established A549, H23 and H460 lung cancer cells. Results of migration and invasion assays revealed that the invasion capability of P1 and A549 cells was higher than that of H23 and H460 cells. However, the migration of P1 cells was similar to that of H23 and H460 cells while A549 demonstrated a superior migrating ability. Western blot analysis indicated that while E-cadherin levels in all lung cancer cells were identified as comparable, P1 cells expressed the highest levels of N-cadherin. In the present study, detachment of cells was demonstrated for the first time to stimulate further transition of E-cadherin to N-cadherin. In addition, this obervation was more pronounced in P1 cells. These observations highlight the importance of EMT in cancer metastasis. In order to study the effect of ethnicity on cancer cell behavior, in the future a large number of Thai patient-derived cell lines must be analyzed.

## Introduction

Epithelial-mesenchymal transition (EMT) is a multistep biological process that enables a normal epithelial cell to possess a mesenchymal phenotype (1). In cancer biology, EMT has received considerable attention since a number of studies have recognized EMT as a hallmark of cancer stemness as well as aggressiveness (2). Alterations in cell behavior caused by EMT, including potentiated migration and increased resistance to apoptosis, have been demonstrated in previous studies (1,2). EMT enables cells to escape interactions and the spatial restrictions imposed by the basement membrane and sustains the viability of the cells when in a detached condition (3,4). Therefore, the transition was previously hypothesized to be associated with the metastatic potential of cancer cells (2,4). Anoikis is a process of cell death which is induced in response to the detachment of the cells from cell-cell and cell-basement interactions. A number of studies have demonstrated that anoikis is a critical process in the inhibition of cancer metastasis in various solid tumors (5,6). In addition, EMT has been demonstrated to be involved in anoikis resistance in melanoma and colon cancer cells (7,8). Downregulation of E-cadherin, together with upregulation of N-cadherin is known to be a key indicator of the EMT process and these proteins are also associated with acquisition of anoikis resistance (2,4,9,10).

Studies on EMT, as well as its association with anoikis resistance in cancer cells originating from Thai individuals, remain limited. In addition, Thailand has a high incidence of lung cancer-related mortalities, the majority of which are associated with cancer metastasis (11). In depth understanding of cancer cell properties is likely to lead to improved precision and efficiency in treating the disease. Therefore, the present study aimed to investigate the expression of the EMT-related markers, E-cadherin and N-cadherin, in cancer cells from a Thai patient. In addition, the correlation of expression levels with anoikis and metastatic characteristics was investigated and compared with those of standard lung cancer cells. These results may improve the development of therapeutic approaches.

## Materials and methods

### Clinical specimen and reagents

Pleural effusions were collected from a 76-year-old male Thai patient with lung adenocarcinoma. Informed consent was obtained from the patient and the study was approved by the ethics committee of the Faculty of Medicine and the ethics committee of the Faculty of Pharmaceutical Sciences (Chulalongkorn University, Bangkok, Thailand). Human non-small cell lung cancer cells, A549, H23 and H460, were obtained from the American Type Culture Collection (Manassas, VA, USA). H23 and H460 cells were cultured in RPMI-1640 medium while A549 cells were cultured in DMEM, supplemented with 10% fetal bovine serum (FBS), 2 mM L-glutamine and 100 U/ml penicillin/streptomycin in a 5% CO_2_ environment at 37°C. Propidium iodide (PI) and Hoechst 33342 were obtained from Sigma-Aldrich (St. Louis, MO, USA). Resazurin-based cell viability reagent (Presto blue) was purchased from Invitrogen Life Technologies (Carlsbad, CA, USA). Specific antibodies against E-cadherin and N-cadherin were obtained from Cell Signaling technology (Danvers, MA, USA) and β-actin antibody was obtained from Santa Cruz Biotechnology (Santa Cruz, CA, USA).

### Specimen preparation

Pleural effusion was centrifuged at 1,600 × g for 10 min at room temperature. The pellet was resuspended with 4 ml sterile balanced salt solution and then centrifuged on a Ficoll gradient (Ficoll-Paque™, GE Healthcare Biosciences, Pittsburgh, PA, USA) at 400 × g for 40 min at 20°C to separate tumor cells from erythrocytes. The layer of mononuclear cells was collected and washed twice with 3 volumes of RPMI medium by centrifuging at 400 × g for 10 min at 20°C. The pellet was then resuspended and the cells were cultured in ACL-4 medium supplemented with 5% FBS at 37°C with 5% CO_2_.

### Anoikis assay

For anoikis evaluation, 6-well tissue culture plates were coated with 200 *μ*l poly 2-hydroxyethylmethacrylate (poly-HEMA; Sigma-Aldrich) and left for 10 h in a laminar flow hood. Cells were seeded in poly-HEMA-coated plates (1×10^5^ cells/ml) and incubated for various times up to 24 h at 37°C. Cell viability was assessed by the addition of 1:50 resazurin for 1 h at 37°C. Fluorescence intensity of resazurin product (resorufin) was measured at 530 nm (excitation wavelength) and 590 nm (emission wavelength) using a microplate reader. Cell viability was calculated as a percentage relative to time zero. All analyses were performed in at least three independent replicate experiments. Apoptosis was determined by Hoechst 33342 DNA fragmentation assay. Briefly, cells were incubated with 10 *μ*g/ml Hoechst 33342 for 30 min and analyzed for apoptosis by scoring the percentage of cells with condensed chromatin and/or fragmented nuclei by fluorescence microscopy (Olympus IX51 with DP70, Olympus, Center Valley, PA, USA).

### Matrigel invasion assay

The invasion assay was performed using Transwell cell culture chambers (Corning Costar No. 3422; Corning, Tewksbury, MA, USA) according to the manufacturer’s instructions with specific modifications. Briefly, polyvinylpyrrolidone-free polycarbonate filters (8.0-mm pore size, Nuclepore Corp., Pleasanton, CA, USA) were pre-coated with 15 *μ*l ice-cold Matrigel (BD Biosciences, Bedford, MA, USA) on the upper surface for 60 min at room temperature. Conditioned medium (500 *μ*l medium with 10% FBS) was added to the lower compartment of the chamber. P1, A549, H23 and H460 cells (5×10^5^) in 1% FBS-containing media were added to the upper compartment of the chamber. Following 48-h incubation, the top side of the insert membrane was scrubbed free of cells with a cotton swab and the bottom side was fixed with ice-cold methanol and stained with Hoechst 33342. Images were captured and scored under a fluorescence microscope (Olympus IX51 with DP70).

### Migration assay

The invasion assay was performed using Transwell cell culture chambers. Conditioned medium (500 *μ*l media with 10% FBS) was added to the lower compartment of the chamber. P1, A549, H23 and H460 cells (5×10^5^) in 1% FBS-containing media were added to the upper compartment of the chamber. Following 12-h incubation, the top side of the insert membrane was scrubbed with a cotton swab and the bottom side was fixed with ice-cold methanol, stained with Hoechst 33342 and scored under a fluorescence microscope (Olympus IX51 with DP70).

### Western blot analysis

Following specific treatments, cells were incubated in lysis buffer containing 20 mM Tris-HCl (pH 7.5), 1% Triton X-100, 150 mM sodium chloride, 10% glycerol, 1 mM sodium orthovanadate, 50 mM sodium fluoride, 100 mM phenylmethylsulfonyl fluoride and a commercial protease inhibitor cocktail (Roche Diagnostics, Basel, Switzerland) for 30 min on ice. Cell lysates were collected and determined for protein content using the Bradford method (Bio-Rad, Hercules, CA, USA). Equal amount of proteins of each sample (40 *μ*g) were denatured by heating at 95°C for 5 min with Laemmli loading buffer and subsequently loaded onto a 10% SDS-polyacrylamide gel to undergo electrophoresis. Following separation, proteins were transferred onto 0.45 *μ*m nitrocellulose membranes (Bio-Rad). The transferred membranes were blocked for 1 h in 5% nonfat dry milk in TBST [25 mM Tris-HCl (pH 7.5), 125 mM NaCl and 0.05% Tween-20] and incubated with the appropriate primary antibodies at 4°C overnight. Membranes were washed twice with TBST for 10 min and incubated with horseradish peroxidase-coupled isotype-specific secondary antibodies for 1 h at room temperature. The immune complexes were detected by enhancement with a chemiluminescence substrate (Supersignal West Pico; Pierce Biotechnology, Inc., Rockford, IL, USA) and quantified using analyst/PC densitometry software (Bio-Rad).

### Statistical analysis

Data are presented as mean ± SD from three or more independent experiments. Statistical analysis was performed by Student’s t test. P<0.05 was considered to indicate a statistically significant difference.

## Results

### Anoikis response of P1, A549, H23 and H460 cells

**Figure 1:**
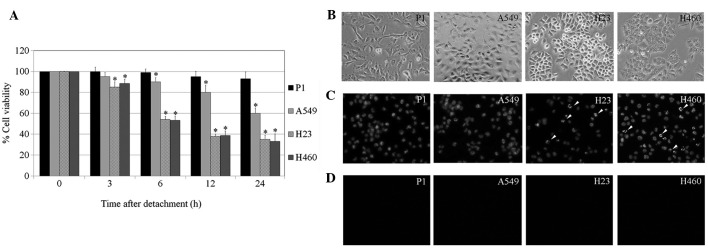
Anoikis response of lung cancer P1, A549, H23 and H460 cells. (A) Cells were detached and suspended in poly-HEMA-coated plates for various time points (0–24 h). Following detachment, cells were collected and survival was determined using the resazurin assay. Viability in each type of detached cells at time 0 was considered as 100%. Columns represent mean ± SD (n=3), ^*^P<0.05, vs. time 0. (B) Phase-contrast micrograph reveals morphology of P1, A549, H23 and H460 cells. (C) Representative images of detached cells assayed for apoptosis by Hoechst 33342 staining following detachment for 12 h. Arrows indicate nuclear condensation and/or fragmentation. (D) Representative images of detached cells assessed for necrosis by PI staining following detachment for 12 h indicating no cells have been stained by PI. HEMA, poly 2-hydroxyethylmethacrylate; PI, propidium iodide.

EMT is associated with the metastatic potential of numerous types of human cancer (12). Knowledge of factors that affect cancer metastasis may benefit the development of novel treatment strategies as well as improve the sensitivity of methods of diagnosis for this life-threatening disease. To investigate the correlation between the metastatic potential of lung cancer cells and EMT, the anoikis response was characterized in primary lung cancer P1 and lung cancer A549, H23 and H460 cells. Cells were detached and cultured in suspended condition over various times. At 0, 3, 6, 12 and 24 h, cell viability was evaluated using the resazurin-based assay. Fig 1 indicates that the detachment-induced apoptosis was significantly suppressed in the primary lung cancer cells which had been isolated from a Thai patient (P1 cells), compared with standard lung cancer cells. A significant reduction in the viability of all lung cancer cell lines, A549, H23 and H460, was detected as early as 6 h, while P1 exhibited non-significant reductions in viability after detachment in the 24 h period. In addition, apoptosis and necrosis were detected in these cells using Hoechst 33342 and PI staining. The results indicate that cell detachment mediated cell death largely through apoptosis, since only a limited number of PI-positive cells were detected (data not shown). As expected, the apoptosis found in P1 populations was extremely low compared with that of other cells. Together, these results indicate that the anoikis-resistance ability of P1 cells may be responsible for their high metastatic potential.

### Migration and invasion of P1, A549, H23 and H460 cells

**Figure 2:**
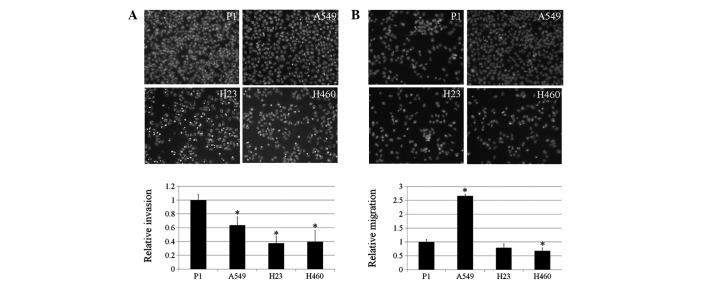
Invasion and migration of P1, A549, H23 and H460 cells. (A) Lung cancer cells were assessed for their invasive characteristics using Matrigel-coated membranes in Transwell chambers. After 48 h, invaded cells were fixed, stained with Hoechst 33342 and visualized under a fluorescence microscope. Columns represent mean ± SD (n=3), ^*^P<0.05, vs. P1 cells. (B) Cells were assessed for migratory capability. Following 12 h, migrated cells were fixed, stained with Hoechst 33342 and visualized under a fluorescence microscope. Columns represent mean ± SD (n=3), ^*^P<0.05, vs. P1 cells.

Enhanced abilities of cancer cells to migrate and invade are hallmarks of advanced stage cancer and aggressiveness (13). In addition, EMT has been linked to the increasing capacity of cancer cells to invade tissues and migrate (2,4). The present study investigated the migration and invasion behaviors of P1, A549, H23 and H460 cells. The results indicate that P1 and A549 cells have significantly enhanced invasion ability compared with H23 and H460 cells (Fig. 2A). For invasion, A549 exhibited the highest ability to migrate in comparison with the P1, H23 and H460 cells. These observations may be useful for understanding the behavior of lung cancer of various origins.

### Switch from E- to N-cadherin in lung cancer cells

It is well accepted that the alteration of cell interactions caused by the decrease of E-cadherin, concomitant with an increase in N-cadherin, is an important indicator of EMT (4, 12). To investigate whether EMT is the underlying mechanism by which P1 cells exhibit an enhanced ability to undergo metastasis, including anoikis resistance and invasion, the present study determined the protein levels of E- and N-cadherin in lung cancer cells.

**Figure 3:**
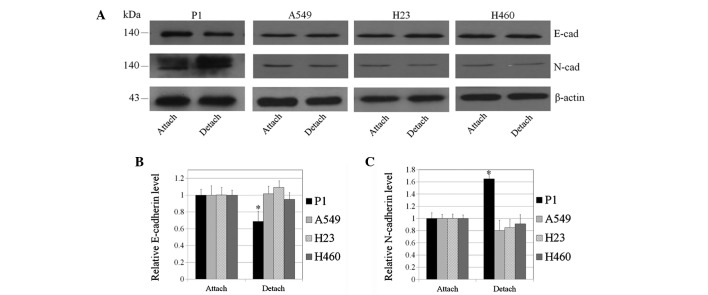
Cadherin switch in P1 cells induced by cell detachment. (A) Lung cancer cells were detached and suspended in poly-HEMA-coated plates for 24 h, cell lysates were collected and E- and N-cadherin expression was analyzed by immunoblotting. β-actin was used as a control. (B) Effect of cell detachment on E-cadherin expression in various lung cancer cells. Columns represent mean ± SD (n=3), ^*^P<0.05, vs. adherent cells. (C) Effect of cell detachment on N-cadherin expression in various lung cancer cells. Columns represent mean ± SD (n=3), ^*^P< 0.05, vs. adherent cells. HEMA, poly 2-hydroxyethylmethacrylate.

Western blot analysis revealed that E-cadherin expression in P1, A549, H23 and H460 cells was comparable (Fig. 3A). The expression level of N-cadherin was enhanced in P1 cells while such an expression was barely detectable in other lung cancer cells. These results indicate that increased EMT of P1 cells may, at least in part, increase the metastatic potential of these cells. Induction of EMT is hypothesized to depend on multiple signals, however, the majority of these signals are unknown. In addition, the effect of cell detachment on EMT was determined by comparing E- and N-cadherin expression in the attached and detached cells. In the present study, cell detachment has been demonstrated to significantly enhance the cadherin switch from E- to N-cadherin in P1 cells (Fig. 3). To a lesser degree, this was also observed in A549, H23 and H460 cells.

## Discussion

EMT is well known to have a significant impact on cancer progression and metastasis (2,4,9,10,12). A number of studies have demonstrated that cancer cells are able to develop a mesenchymal phenotype which enhances their malignancy (7,14). One characteristic of mesenchymal cells is the ability to survive in suspended conditions which may enable cancer cells to undergo metastasis (2,4,9,10). In addition, reduced interaction of cells with the basement membrane during EMT may facilitate the dissemination of cancer cells from their tumors of origin (4).

Lung cancer in Thailand has become a significant cause of cancer-related mortality (11), with the majority of such mortalities due to cancer metastasis (11, 15). In our previous study of cancer cells from various ethnicities, primary lung cancer P1 cells were demonstrated to exhibit sufficient cisplatin resistance, together with characteristics of cancer (16). The present study revealed that the degree of EMT in P1 was high in comparison with that of lung cancer cells, namely A549, H23 and H460 cells. Although the downregulation of E-cadherin in P1 was not intense, the marked increase in levels of N-cadherin represented EMT in the P1 cells. Consistent with previous studies reporting that EMT enhances anoikis resistance in numerous cells (2,4,7), the transition of E- to N-cadherin was demonstrated in the present study to be tightly associated with the ability to resist anoikis in P1 cells.

A number of studies explaining the involvement of cancer metastasis and the process of EMT have reported that EMT enables cancer cells to migrate away from the tumour of origin (2,4,7,9). However, it is unclear whether the process of cell detachment triggers EMT further. In the present study, lung cancer P1 cells were identified to exhibit an increased expression of N-cadherin, concomitant with decreased E-cadherin levels, following detachment. This observation supports the hypothesis reporting a link between EMT and the metastatic process. Not all cancer cells have been identified to undergo EMT (17), therefore, cells that possess the potential to carry out this transition may have a greater probability of metastasizing successfully.

With regard to migration and invasion, certain studies have correlated such abilities with EMT (4,7,8,9). However, in the current study, P1 cells only were observed to exhibit an increased invasion capability, compared with other cancer cells. In addition, A549 cells, which exhibited the most significant ability to migrate and invade, revealed minimal EMT. These results indicate that EMT in P1 cells may regulate invasion and migration to a lesser extent compared with the anoikis response.

Based on these observations, the ability of cancer cells to undergo EMT may potentiate the metastasis of lung cancer cells. In addition, the present study indicates that ethnicity may affect EMT and may facilitate an improved understanding of cancer cell biology.
